# Characterization of the urinary microbiome in healthy dogs

**DOI:** 10.1371/journal.pone.0177783

**Published:** 2017-05-17

**Authors:** Erin N. Burton, Leah A. Cohn, Carol N. Reinero, Hans Rindt, Stephen G. Moore, Aaron C. Ericsson

**Affiliations:** 1 Department of Veterinary Pathobiology, University of Missouri College of Veterinary Medicine, Columbia, Missouri, United States of America; 2 Department of Veterinary Medicine and Surgery, University of Missouri College of Veterinary Medicine, Columbia, Missouri, United States of America; 3 University of Missouri Metagenomics Center (MUMC), Columbia, Missouri, United States of America; 4 Division of Animal Sciences, University of Missouri College of Agriculture, Food and Natural Resources, Columbia, Missouri, United States of America; University of North Texas, UNITED STATES

## Abstract

The urinary bladder in healthy dogs has dogmatically been considered free of bacteria. This study used culture independent techniques to characterize the healthy canine urinary microbiota. Urine samples collected by antepubic cystocentesis from dogs without urinary infection were used for DNA extraction. Genital tract and rectal samples were collected simultaneously from the same dogs. The V4 hypervariable region of the 16S rRNA bacterial gene was amplified and compared against Greengenes database for OTU assignment and relative abundance for urine, genital, and rectal samples. After excluding 4 dogs with cultivable bacteria, samples from 10 male (M; 1 intact) and 10 female (F) spayed dogs remained. All samples provided adequate genetic material for analysis. Four taxa (*Pseudomonas* sp., *Acinetobacter* sp., *Sphingobium* sp. and *Bradyrhizobiaceae*) dominated the urinary microbiota in all dogs of both sexes. These taxa were also detected in the genital swabs of both sexes, while the rectal microbiota differed substantially from the other sample sites. Principal component (PC) analysis of PC1 through PC3 showed overlap of urinary and genital microbiota and a clear separation of rectal swabs from the other sample sites along PC1, which explained 44.94% variation. Surprisingly, the urinary microbiota (mean # OTU 92.6 F, 90.2 M) was significantly richer than the genital (67.8 F, 66.6 M) or rectal microbiota (68.3 F, 71.2 M) (p < 0.0001), with no difference between sexes at any sample site. The canine urinary bladder is not a sterile environment and possesses its own unique and diverse microbiota compared to the rectal and genital microbiota. There was no difference between the sexes at any microbiota sample site (urine, genital, and rectal). The predominant bacterial genus for either sex in the urine and genital tracts was *Pseudomonas* sp.

## Introduction

Over the past decade there has been increasing scientific evidence, in both humans and domestic species, supporting the important role of an individual’s microbiome on health and wellness. While the majority of studies in both human and veterinary medicine have focused on the gastrointestinal microbiome, rich, site-specific bacterial communities have also been documented in other tissues previously considered to be sterile[1–5].With the advent of extremely sensitive culture-independent methods of characterizing complex microbial communities (e.g., metagenomics and 16S rRNA sequencing), evaluation of these microbial communities is increasingly feasible. These methods allow for the identification of specific bacterial, archaeal, fungal, and viral strains, even in instances of minimal colonization[6]. In both human and veterinary medicine, targeted 16S rRNA amplicon sequencing has been used extensively to characterize the gastrointestinal microbiota (GM)[7–10]. More recently, characterization of the human urinary microbiome (UM) has been described, mostly in women, using various collection methods including midstream voided, suprapubic aspiration (SPA), and transurethral catheterization (TUC) techniques and various microbial community characterization techniques (routine culture, enhanced quantitative urine culture (EQUC) and/or 16S rRNA sequencing)[1–3, 5, 11–22]. Wolfe et. al. (2012) were the first to use early 16S rRNA sequencing techniques to characterize the urinary bladder microbes in TUC- and SPA-collected urine samples from women without urinary symptoms. These SPA- and TUC-collected samples revealed DNA evidence of rich, diverse, and living microbial populations[19]. Later, the same investigators used an EQUC protocol to demonstrate that these bacteria were alive[1]. This and other studies have shown that routine urine culture is insensitive for detection of most bacterial species found in the urogenital tract including uropathogens[1, 3, 19, 22].

Given the impact of the GM on gastrointestinal health, it is likely that the UM impacts urinary health. There may well be a “core” UM which, when disrupted, contributes to disease[3, 5, 23–28]. Perhaps similar strategies to those used to beneficially modulate gastrointestinal dysbiosis could be used to correct urinary dysbiosis for the prevention or treatment of urinary tract infection (UTI) or other causes of cystitis[29].

UTIs are a common problem in dogs, with an estimated 14% of all dogs experiencing a routine culture-positive UTI in their lifetime[30]. Yet, to the authors’ knowledge, studies evaluating the presence or composition of the urinary microbiome of healthy dogs have not been performed. The aims of this study were to identify and describe the urinary microbiome of healthy, routine urine culture-negative adult dogs of either sex, and to evaluate if the core microbiota of the healthy canine urinary bladder is similar or related to the genital microbiome or GM.

## Materials and methods

### Population

A population of dogs undergoing medical procedures requiring sedation or anesthesia at the University of Missouri Veterinary Health Center was used with fully informed owner consent. Urine, genital (preputial or vaginal) swabs, and rectal swabs were collected from equal numbers of male and female dogs weighing ≥ 15 kg, and between 1 and 10 years of age. Samples were not collected from dogs that had received antibiotics, probiotics, or corticosteroids within the previous 30 days, had received intravenous or subcutaneous fluids therapy within the previous 24 hours, demonstrated any evidence of systemic infection (including severe periodontal disease), or had any history of clinical signs associated with urinary disease (e.g., dysuria, pollakiuria, stranguria, gross hematuria). Urinalysis findings of pyuria (>5 WBC/ hpf) or bacteriuria, or bacterial growth on routine urine culture after sampling resulted in exclusion from further analysis. All animal use was approved by the University of Missouri Institutional Animal Care and Use Committee, under protocol #8270.

### Sample collection

To minimize any discomfort, all but 2 samples were collected under sedation or general anesthesia prior to unrelated planned medical procedures. Urine was collected from all dogs via antepubic cystocentesis using a 22 ga. needle; aliquots were used for routine urinalysis (5 mL), routine urine culture (1 mL), and 16S rRNA amplicon sequencing (30 mL). To decrease dermal microbiota contamination of the urine samples, the collection site was disinfected with 70% isopropyl alcohol. Additionally, the collection needle was discarded and a sterile needle was placed on the end of the syringe to minimize dermal microbiota transfer to the aliquots. A sterile, moistened cotton tip applicator was inserted into the vagina to approximately the level of the vaginal vault or into the preputial sheath to the level of the glans penis and swabbed vigorously for 15 to 20 seconds to collect a genital sample. Another sterile, moistened cotton tip applicator was inserted approximately 1 inch into the rectum and swabbed vigorously for 15 to 20 seconds for the rectal sample. These swabs were placed into separate 15 mL conical vials, each containing 5 mL of sterile water. Urine, genital, and rectal swabs for 16S rRNA amplification were centrifuged at 2150 × g for 20 minutes. The supernatant was discarded before 800 μL of lysis buffer was added to the remaining pellet and vortexed until thoroughly mixed. The mixtures were transferred to 2.0 mL sterile round bottom tubes and stored at -80°C until DNA extraction.

### DNA extraction

DNA from urine, feces, and genital swabs were manually extracted using an adaptation of a published technique[31]. The adaptations include bead beating with a single sterile 0.5 cm-diameter stainless steel bead rather than zirconia beads, and the continuous processing of each sample as a single aliquot with no need to split the sample during precipitation to accommodate the larger sample volume as reported by Yu et. al[31]. Purity of DNA was assessed via spectrophotometry (Nanodrop, Thermo Fisher Scientific, Waltham, MA); yield was determined via fluorometry (Qubit, Life Technologies, Carlsbad, CA) using Qubit dsDNA BR assay kits (Life Technologies).

### 16S rRNA library preparation, sequencing, and informatics analysis

Extracted fecal, urine, and genital DNA was processed at the University of Missouri DNA Core Facility. Bacterial 16S rRNA amplicons were generated via amplification of the V4 hypervariable region of the 16S rRNA gene using single-indexed universal primers (U515F/806R) flanked by Illumina standard adapter sequences and the following parameters: 98^°^C^(3:00)^ + [98^°^C^(0:15)^ + 50^°^C^(0:30)^ + 72^°^C^(0:30)^] × 25 cycles + 72^°^C^(7:00)^. Amplicons were then pooled for sequencing using the Illumina MiSeq platform and V2 chemistry with 2× 250 bp paired-end reads, as previously described[7].

Briefly, contiguous DNA sequences were assembled using FLASH software[31], and culled if found to be short after trimming for a base quality less than 31. Qiime v1.8[32, 33] software was used to perform *de novo* and reference-based chimera detection and removal, and remaining contiguous sequences were assigned to operational taxonomic units (OTUs) via *de novo* OTU clustering and a criterion of 97% nucleotide identity. Taxonomy was assigned to selected OTUs using BLAST[34] against the Greengenes database[35] of 16S rRNA sequences and taxonomy. Principal component analysis was performed with ¼ root-transformed OTU relative abundance data using a non-linear iterative partial least squares algorithm, implemented in an open access Excel macro available from the Riken Institute (http://prime.psc.riken.jp/Metabolomics_Software/). All data have been deposited in the NCBI Sequence Read Archive (SRA) under BioProject identification PRJNA379615.

### Urine culture

Samples intended for routine urine culture were processed by the Veterinary Medical Diagnostic Laboratory Bacteriology section. Briefly, using calibrated loops and a filter, 1/100 and 1/1000 of a single mL of urine was delivered to both blood agar and MacConkey agar plates for aerobic bacterial isolation. Additionally, 1/100 of a single mL of urine was delivered to an anaerobic blood agar plate. Plates were incubated under routine culture conditions for 72 hours and examined for colony formation. Any sample with cultivable bacteria was excluded from microbiome analysis.

### Statistical analysis

Mixed model procedures were implemented in SAS (SAS Institute, 2006) to determine the effect of sex and sample site on richness, Chao1, and Shannon diversity indices. Sex, sample site, and their interaction were included as fixed effects and animal nested within sex was included as a random effect. Within-animal comparison of Bray-Curtis distances between urine microbial profiles and either matched rectal or genital communities was performed via Wilcoxon signed rank test using SigmaPlot 12.3 (Systat Software Inc., San Jose, CA). Testing for main effects of sample site and sex on microbial composition (at the level of OTU), as well as interactions between fixed variables, was performed via two-way PERMANOVA of ranked Bray-Curtis distances using Past 3.13 software[36]. For all tests, *p* < 0.05 was considered significant.

## Results

Samples were collected from a total of 24 dogs. Eleven dogs were undergoing orthopedic procedures, with neutering, ophthalmologic, and imaging procedures accounting for the remainder of the sedated or anesthetized dogs. Samples were also obtained from two well-behaved un-sedated dogs presented for wellness examinations. Due to bacterial growth on routine culture, 4 samples were excluded from further analysis, leaving 10 samples each from female (all spayed) and male (9 castrated, 1 intact) dogs. The mean age of included dogs was 4.75 years (range 1 to 9), with a mean body weight of 32.1 kg (range 17.4 to 67.5) (Table 1). Sequencing of 16S rRNA amplicon libraries generated a mean (± SEM) of 18965 (± 1590), 13112 (± 2886), and 121026 (± 11383) reads from urine, genital swab, and rectal swab samples respectively. While no negative controls were included in the current analysis, periodic analysis of negative reagent controls in our lab has consistently yielded zero to < 500 reads per sample. Thus, the resulting read counts suggest true colonization.

**Table 1:** Population study demographic.

Sex	Breed	Age (Years)	Weight (Kg)	Presentation
MI	Mixed	1	27.6	Castration
MC	Mixed	2	46.5	Orthopedic
MC	Lab. Ret.	3	34.6	Orthopedic
MC	Mixed	6	40	Orthopedic
MC	Boxer	4	34.9	Orthopedic
MC	Amer. Staf.	6	32	Orthopedic
MC	GSD	5	38.8	Nasal Computed Tomographic Scan
MC	Aust. Shep.	4	18.1	Soft Tissue Injury
MC	Rottweiler	5	67.5	Orthopedic
MC	Lab. Ret.	4	27.8	Orthopedic
FS	Amer. Staf.	6	23.3	Orthopedic
FS	Aust. Shep.	6	26.2	Orthopedic
FS	Mixed	1	31.3	Orthopedic
FS	Lab. Ret.	9	37.2	Cataract Surgery
FS	Border Collie	4	17.4	Wellness Exam
FS	Lab. Ret.	6	36.2	Orthopedic
FS	Amer. Staf.	4	23.3	Orthopedic
FS	Mixed	5	36.5	Orthopedic
FS	Aust. Shep.	7	20.5	Wellness Exam
FS	Aust. Shep.	7	21.8	Wellness Exam

^a.^ Lab. Ret. = Labrador Retriever, Amer. Staf. = American Staffordshire Terrier, Aust. Shep. = Australian Shepard, GSD = German Shepard Dog, MC = Male castrated, MI = Male intact, FS = Female spayed

### Diversity and richness of the canine urinary, genital, and fecal microbiota

**Fig 1:**
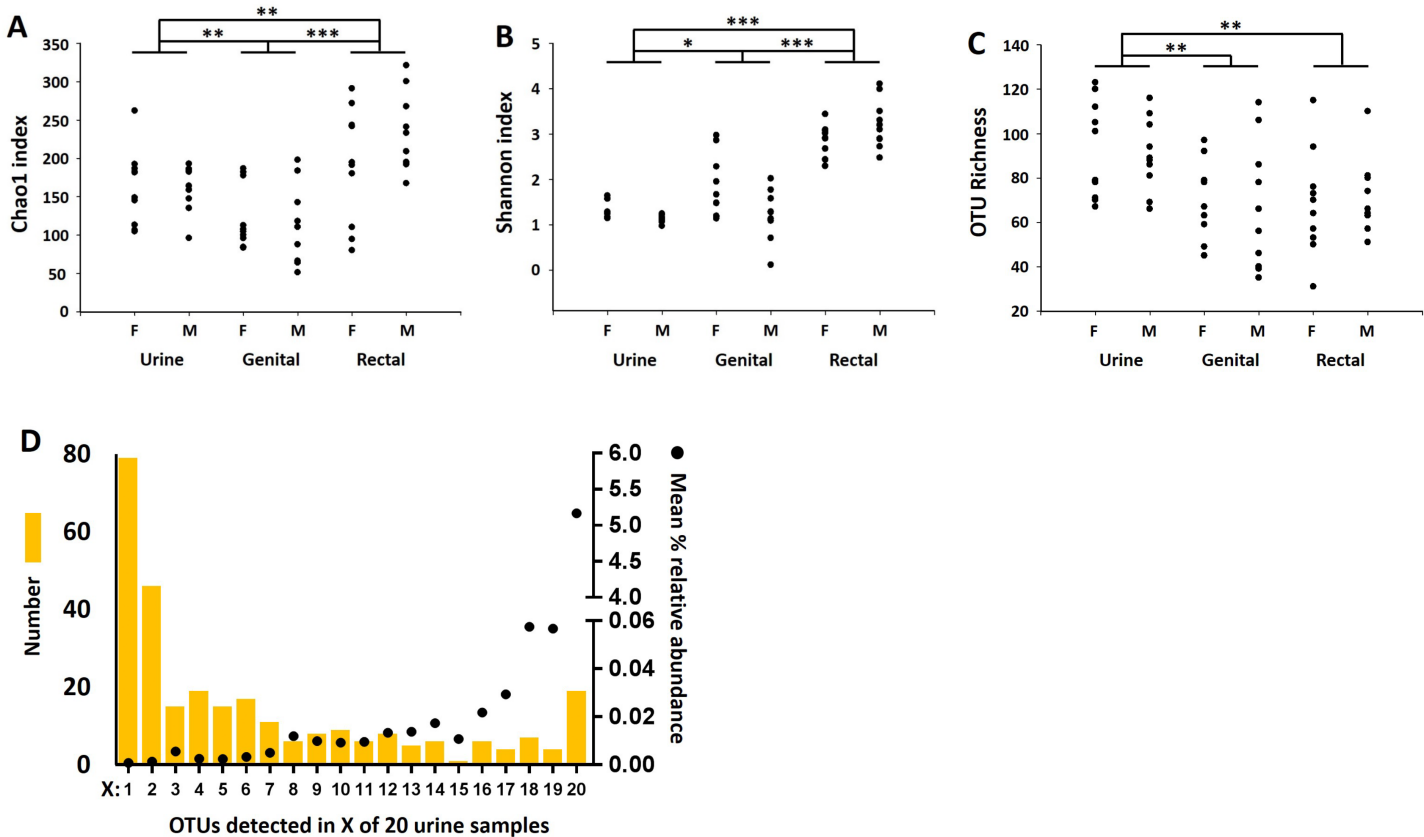
Dot plots showing Chao1 indices (A), Shannon indices (B), and operational taxonomic unit (OTU) richness (C) of microbiota detected in the urine samples, genital swabs, and rectal swabs, collected from 20 healthy female (F, *n* = 10) and male (M, *n* = 10) dogs; bar chart showing the number of OTUs detected in X out of 20 urine samples, overlaid with dots indicating the mean relative abundance of those OTUs, demonstrating the high sparsity of the urinary microbiota. *p<0.05, **p<0.01, ***p<0.001.

Richness is an indicator of the overall number of different taxa present in a sample regardless of distribution; α-diversity is an indicator of the combined richness and evenness of distribution among the various taxa detected in a sample, with greater evenness resulting in greater α-diversity. That said, α-diversity can be calculated several different ways with differential weight placed on the richness or evenness. Two commonly used metrics of α-diversity were compared between groups, yielding slightly different results. Comparison of Chao1 indices, which preferentially weights the richness of a sample based on the numbers of singletons and doubletons (i.e., sequences detected only once or twice in a given sample, respectively), detected a significant main effect of sample site (*p* < 0.0001; F = 16.59), with rectal and genital swab samples harboring the greatest and lowest numbers of distinct sequences respectively (**Fig 1A**). No significant effect of sex on the Chao1 index was detected. Similarly, testing of the Shannon diversity index, a more traditional measure of α-diversity which places more weight on the evenness of taxa, revealed a main effect of sample site (*p* < 0.0001; F = 94.91), as well as a significant interaction (*p* = 0.0036; F = 6.27) between sample site and sex (**Fig 1B**). Similar to the Chao1 index, there was no main effect of sex on the Shannon diversity index. Despite the appreciably lower coverage of urine and genital swab samples reflecting the low biomass of those samples, the overall OTU richness (i.e., number of distinct OTUs detected) of urine samples demonstrated a significant main effect of sample site (**Fig 1C**, *p* < 0.0001; F = 23.07), with urine samples harboring, on average, over 20 more OTUs than either of the other sample sites. Again, there was no main effect of sex on richness (*p* = 0.480; F = 0.51). Lastly, the microbial profile generated from urine samples represented extremely sparse datasets; that is, a high proportion of taxa were detected at very low relative abundance and in a limited number of individual urine samples (**Fig 1D**).

### Composition of the canine urinary microbiome

**Fig 2:**
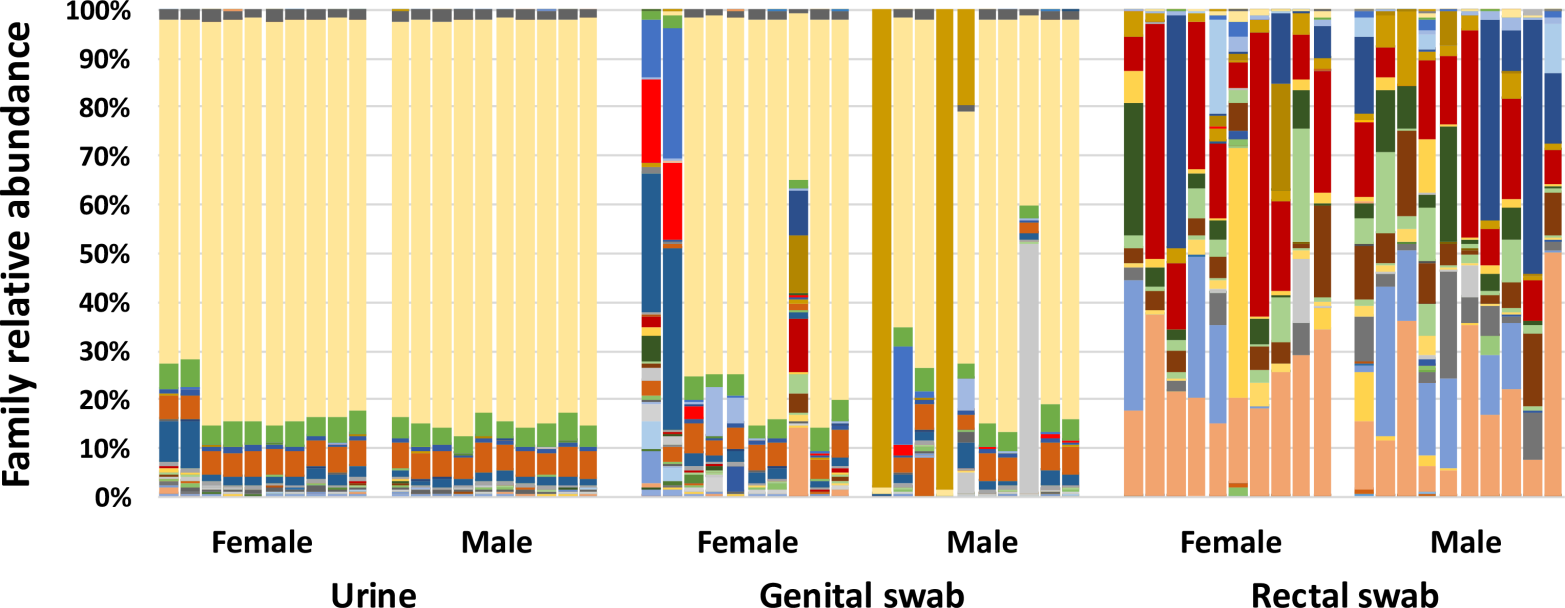
Stacked bar charts showing relative abundance of microbial DNA detected via 16S rRNA amplicon sequencing and annotated to the taxonomic level of family, in samples collected via cystocentesis (urine), vaginal or preputial swab (genital swab), or rectal swab from 20 healthy adult dogs (*n* = 10 female, 10 male).

Similar UM profiles were observed in both sexes, particularly with regard to the dominant OTUs detected. Only five OTUs, all within the phylum *Proteobacteria*, were detected at greater than 1% mean relative abundance in all urine samples. These included *Pseudomonas* sp. (mean ± SD of 81.15 ± 0.89%), *Sphingobium* sp. (4.70 ± 0.09%), *Acinetobacter johnsonii* (4.54 ± 0.17%), and unclassified (UC) microbes in the families *Bradyrhizobiaceae* (2.40 ± 0.53%) and UC family *Xanthomonadaceae* (1.95 ± 0.04%) (**Fig 2**). An additional nine OTUs were detected in urine samples at greater than 0.1% mean relative abundance, including *Delftia* sp., UC order *Streptophyta*, *Sphingomonas* sp., *Brevundimonas diminuta*, UC family *Caulobacteraceae*, *Propionibacterium acnes*, *Pedobacter* sp., *Staphylococcus* sp., and *Bacteroides* sp. Of the 14 OTUs listed above, all were detected in all 20 urine samples with the exception of *Bacteroides* sp. which was detected in 19 of 20 samples. An additional six OTUs were detected in all 20 urine samples, albeit at extremely low mean relative abundance, including UC family *Pseudomonadaceae* (mean 0.09%), *Streptococcus* sp. (0.08%), UC family *Sphingomonadaceae* (0.07%), *Agrobacterium* sp. (0.06%), *Acinetobacter* sp. (0.04), and UC family *Methylobacteriaceae* (0.03%).

### Comparison of urinary microbiome to genital and fecal microbiota

The five most common taxa observed in the UM were also detected in the majority of genital swabs of both sexes. Similar to the UM, *Pseudomonas* sp. was the dominant OTU detected in 8 and 7 of 10 female and male genital swabs, respectively (**Fig 2**). Notably, the other 2 female genital swabs, collected from two dogs from the same household, yielded substantial relative abundances of UC family *Bradyrhizobiaceae*, *Conchiformibius* sp., and UC family *Pasteurellaceae*. The vaginal swab from a third female dog harbored relatively high proportions of several typical canine fecal microbes including members of the families *Bacteroidaceae*, *Lachnospiraceae*, *Ruminococcaceae*, *Fusobacteriaceae*, *Helicobacteraceae*, and *Campylobacteraceae*. In the other three male genital swabs, *Pseudomonas* sp., *Mycoplasma* sp. (2 of 10 preputial swabs) and *Streptococcus* sp. (1 of 10 preputial swabs) were the dominant OTUs. A comparison of the Bray-Curtis distances between the urine sample and either rectal or genital swab samples within each dog revealed a significant difference (p < 0.001, t-test) with mean ± SD urine:genital swab distances of 0.54 ± 0.12 and urine:rectal swab distances of 0.25 ± 0.09. These results thus demonstrate that the urinary microbiota was more similar in composition to the communities detected in the genital swab samples, relative to the fecal microbiota.

Not surprisingly, the rectal microbiota differed substantially from the urinary and genital sample sites. In agreement with previous reports, dominant OTUs included *Fusobacterium* sp. (mean ± SEM relative abundance of 18.44 ± 3.46%), *Bacteroides* sp. (15.00 ± 1.96%), *Helicobacter* sp. (9.83 ± 3.76%), *Prevotella copri* (9.16 ± 2.48%), *Bacteroides plebeius* (5.37 ± 1.38%), *Megamonas* sp. (4.39 ± 1.69%), *Faecalibacterium prausnitzii* (3.93 ± 1.29%), *Prevotella* sp. (3.92 ± 1.16%), *Sutterella* sp. (3.30 ± 0.73%), *Porphyromonas* sp. (3.21 ± 2.53%), *Roseburia* sp. (2.10 ± 0.84%), UC family *Lachnospiraceae* (1.80 ± 0.52%), *Anaerobiospirillum* sp. (1.74 ± 1.05%), *Campylobacter* sp. (1.72 ± 1.11%), UC order *Clostridiales* (1.43 ± 0.29%), *Turicibacter* sp. (1.11 ± 0.72%), *Bacteroides ovatus* (1.08 ± 0.49%), and *Phascolarctobacterium* sp. (1.03 ± 0.26%). All other OTUs detected in rectal swabs were present at below 1% mean relative abundance.

**Fig 3:**
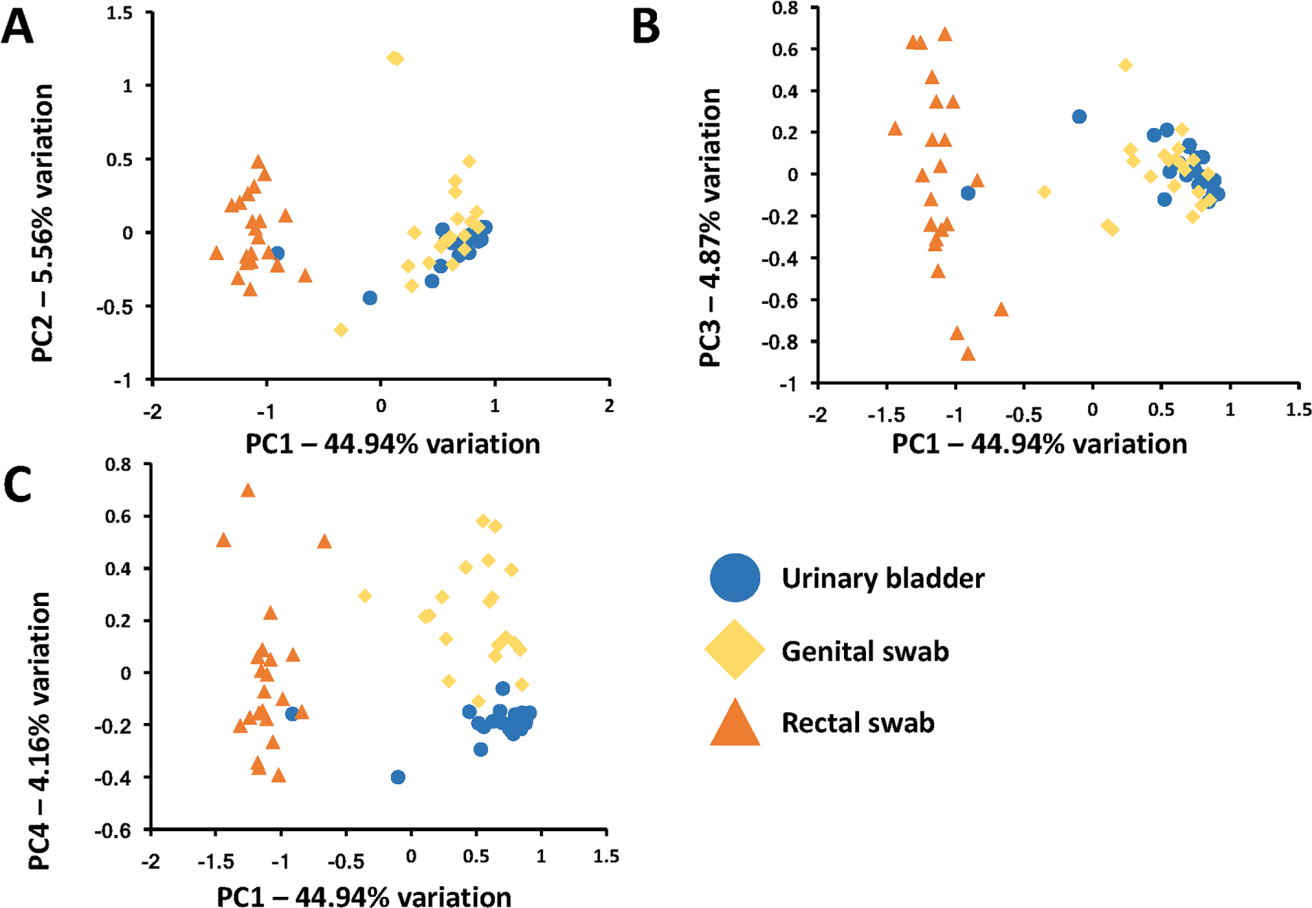
Principal component analysis of urine, genital swab, and rectal swab microbiota, as determined via 16S rRNA amplicon sequencing, colored by sample collection site, from 20 healthy adult dogs (*n* = 10 female, 10 male). Plots depict PC1 versus PC2 (**A**), PC1 versus PC3 (**B**), and PC1 versus PC4 (**C**).

**Fig 4:**
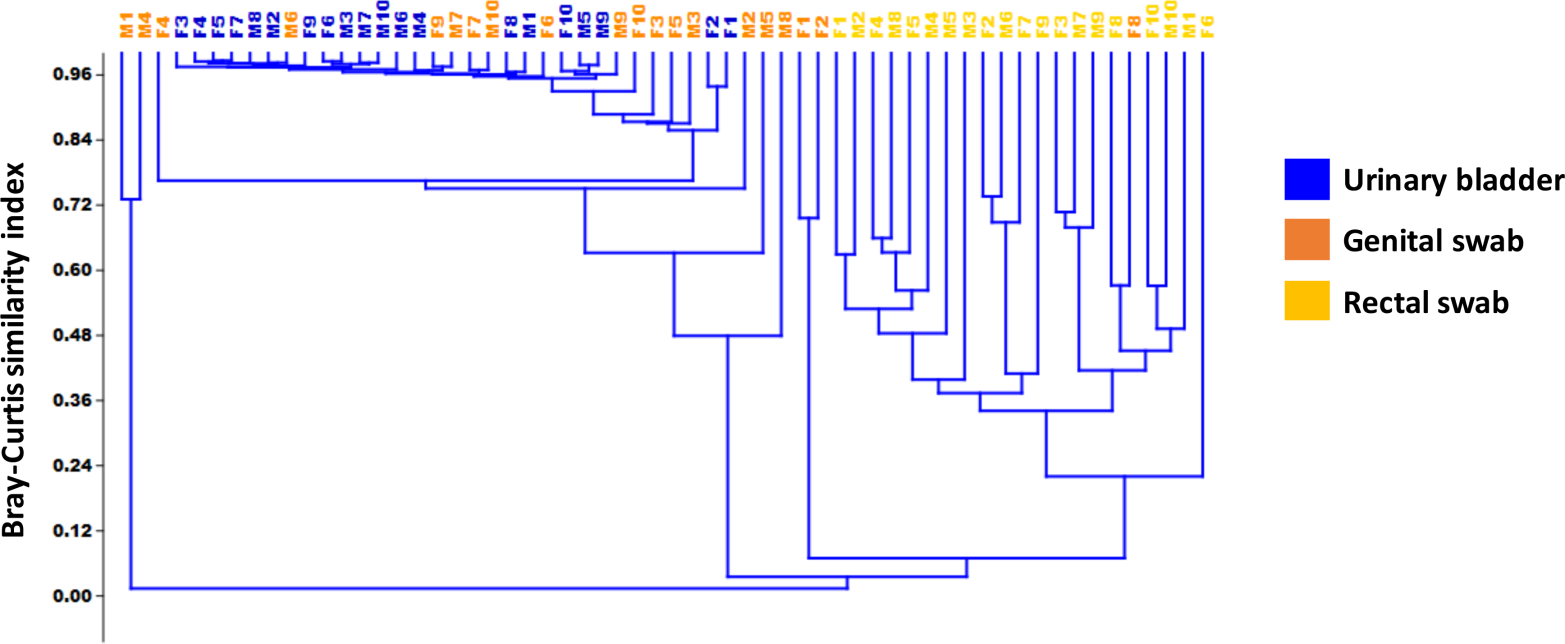
Agglomerative hierarchical clustering analysis of Bray-Curtis similarity indices, performed via unweighted pair group method with arithmetic mean (UPGMA), legend at right.

To more accurately assess relatedness of samples from the three different sample sites, principal component analysis (PCA) and hierarchical clustering were performed. PCA showed clear separation of rectal swabs from the other sample sites along PC1, which explained 44.94% variation (**Fig 3A**). Principal components 1, 2, and 3 between the genital swabs and urine samples demonstrate that there is considerable overlap between these sites potentially indicating intermingling of microbes between the two sample sites. (**Fig 3A and 3B**). PC4 however, capturing 4.16% variation, provided clear separation of genital swab and urine samples (**Fig 3C**). As a confirmatory visualization, agglomerative hierarchical clustering analysis was performed. Reminiscent of the comparisons of PC1, PC2, and PC3, the rectal swab samples formed a distinct branch of the dendrogram, while urine and genital swab samples are intermingled (**Fig 4**). However, all but two urine samples fell within the most distal (uppermost) portion of the dendrogram reflecting the high compositional uniformity of those samples. Testing via two-way PERMANOVA of Bray-Curtis distances detected a significant main effect of sample site (*p* < 0.0001) but no significant main effect of sex (*p* = 0.3188) and no significant interactions (*p* = 0.3965). To allow for pairwise comparisons, male and female samples were thus pooled and compared between sample sites using one-way PERMANOVA. All pairwise comparisons yielded *p* values of 0.0001, indicating that the microbial communities detected at each sample site were significantly different in composition.

## Discussion

Outside of routine preventative health care, bacterial cystitis is one of the most common reasons dog owners seek veterinary care with estimates that 14% of dogs will suffer a UTI during their lifetime[30, 37, 38]. Historically, the identification of bacteria or fungi in urine aseptically obtained from the bladder and processed by routine urine culture methods has defined UTI. It has been widely accepted that negative bacterial culture signified bladder sterility. While this method continues to be the gold standard for the diagnosis of clinically relevant UTI in dogs, it should be recognized that the treatment and resolution of UTI is likely a more complex process than simply elimination of bacteria identified using only routine anaerobic and aerobic urine cultures[1, 5, 22]. To our knowledge, our laboratory is the first to document the presence of urinary bacterial microbes in dogs using the sequencing of 16S rRNA amplicons. Despite the low overall biomass of the urine samples, the coverage documented by the urinary samples (mean 18965 ± 1590 reads) was similar to the coverage from the genital samples (mean 13112 ± 2886 reads), a site known to be colonized with microbes in health. The urine samples demonstrated a large number of distinct OTUs, described as the sample richness. Thus, we were able to characterize the UM in healthy dogs using a method of detection far more sensitive than routine bacterial culture.

Only five OTUs, all within the phylum *Proteobacteria*, were detected at greater than 1% mean relative abundance in all urine samples. These Gram-negative bacteria included *Pseudomonas* sp., *Sphingobium* sp., *Acinetobacter johnsonii*, and unclassified microbes in the families *Bradyrhizobiaceae*, and *Xanthomonadaceae* (**Fig 2).** Our study only included dogs free from urinary signs, and from which routine culture was negative. However, it is worth noting that the most commonly identified pathogens in clinical urinary tract infection in dogs include *E*. *coli*, *Staphylococcus* spp., *Enterococcus* spp., *Streptococcus* spp., *Proteus* spp., and *Klebsiella* spp.[39–42]. Of these, *Escherichia coli*, *Proteus*, and *Klebsiella* are part of the phylum *Proteobacteria*, while *Staphylococcus*, *Enterococcus*, and *Streptococcus* are instead found within the phylum *Firmicutes*.

*Pseudomonas* sp. and *Acinetobacter* sp. were the two most prevalent bacteria found in the genital tracts of both male and female dogs. Interestingly, previous studies using routine culture methods to characterize the vaginal flora in bitches yielded *E*. *coli* and *S*. *pseudintermedius* as the most common isolates[43]. This represents an entirely different microbiota than observed in our study, further highlighting the limitations of using routine urine culture alone to characterize bacterial populations. In the present study, the identified genital microbiome was similar to the UM in that both shared *Pseudomonas* sp. and *Acinetobacter* sp. as the most abundant taxa (p < 0.001, t-test). While PCA demonstrated marked overlap between the microbiome of these sites, there was also clear separation between the two microbial populations **(Fig 3C).**

There was little overlap between the genital or UM profiles and the fecal microbiome **(Fig 3)**. The five dominant bacteria found in the fecal microbiome included *Fusobacterium* sp., *Bacteroides* sp., *Helicobacter* sp., *Prevotella copri*, and *Bacteroides plebeius*. These findings largely mirror other veterinary studies that have found members of the phyla *Fusobacterium*, *Bacteroidetes*, *Proteobacteria*, and *Firmicutes* predominating the fecal microbiome using 16S rRNA sequencing techniques [4, 8, 10, 44] While no single member of the phyla *Firmicutes* are included in the five most predominant taxa, several members including *Megamonas* sp., *Faecalibacterium prausnitzii*, *Roseburia* sp., UC family *Lachnospiraceae*, and UC order *Clostridiales* were present above 1% mean relative abundance.

Bitches account for the large majority of UTI in dogs but the UM of bitches did not differ from that of male dogs [38]. We found no statistical difference between males and females in the richness, diversity, or composition of the urinary microbiota (*p* = 0.32). This differs from the human literature, where the UM does differ by sex. In women, the predominant genera are *Lactobacillus* and *Gardnerella* with *Streptococcus*, *Staphylococcus*, and *Corynebacterium* following[3, 20, 24–26]. The most common microbes in female urine are also considered the predominant bacterial taxa of the vagina, thus there may be some relationship between the two microbial communities, and urine collection technique (i.e., voided samples) will influence microbes identified[3, 24, 27, 28, 45–47]. In men, *Lactobacillus*, *Streptococcus*, *Ureaplasma*, *Mycoplasma*, *Sneathia*, and several other genera comprise the UM in health[11, 14]. Although the UM and the genital microbiota reported here were unique, there was considerable overlap between the two niches, just as has been described in men and women[2, 12]. Genital and UM may each be colonized by microbes of the mucosal and epidermal surfaces of the vagina or prepuce, bladder wall, or combination thereof.

Although the UM did not differ between the sexes in our study, anatomic differences might explain why bitches are more likely to develop UTI than male dogs. Anatomical anomalies such as hooded vulva, vestibule vaginal stenosis, and ectopic ureters have been reported in female dogs as potential risk factors for development of recurrent or persistent UTI[48]. Additionally, multiple authors speculate that the shorter urethra of female dogs as compared to male dogs results in easier ascent of fecal pathobionts to the urinary bladder. In our study, PCA and hierarchical clustering demonstrated clear separation between the microbiome of collected fecal swabs and the more similar yet distinct genital and urinary samples **(Fig 3)**. This suggests that the fecal microbiome has a less profound influence on the UM in healthy dogs. The most important routes and risk factors for colonization and overt infection deserve further study.

In the present study, 4 dogs without owner-reported clinical signs of UTI were excluded due to a positive routine urine culture. These 4 dogs were found to be culture-positive for *E*. *coli* (2 dogs) or *S*. *pseudintermedius* (2 dogs). While asymptomatic bacteriuria has long been recognized in human medicine, only recently has the concept been translated to veterinary medicine. Of course, dogs cannot report symptoms such as discomfort, and owner observation of the presence or absence of urinary signs may be unreliable. In the past, if bacteria were isolated by routine urine culture methods obtained by sterile cystocentesis, then UTI was said to be present regardless of the presence or absence of clinical signs [37]. Veterinarians have only recently recognized that cultivable bacteria may be isolated from the urine of between 2 and 9% of dogs that are otherwise healthy based on owner history and examination[49, 50]. Further studies are necessary to characterize the clinical course of dogs without signs of urinary illness but with cultivable urinary microbes, as well as to characterize the UM in these “healthy” dogs.

There are limitations to our study. In retrospect, it would have been ideal to obtain control samples from needle puncture of the skin to assure that cross contamination from the skin did not influence our results. However, not only did the technique itself (i.e., replacement of the skin puncture needle prior to dispersion of the urine from the collection syringe after cystocentesis) minimize the risk of cross contamination by skin microbes, but the composition of the UM identified differed markedly from the dermal microbiome previously identified in dogs[51–54]. Another limitation is that the UM, as characterized by PCR-based techniques used in our study, may include non-viable organisms. In fact, dogs with viable bacteria identified on routine culture were excluded from our study. EQUC employing larger volumes of urine plated on a variety of culture media and under different environmental conditions have demonstrated in women that both 1) routine culture methods are extremely insensitive for the detection of microbes, and 2) the diverse UM detected via PCR techniques includes a wide variety of viable, living microbes[1, 3, 22]. The utility of EQUC for identification of viable urinary microbes in dogs is worth exploration.

In recent years, more dogs have been identified with recurrent or refractory UTI[43]. This may be in part related to dogs living longer with chronic conditions that predispose to UTI, such as immunosuppressive states, endocrinopathy, or paraparesis. Repeated bouts of UTI are a source of frustration for owners and veterinarians, with few new interventions to ameliorate this problem. Further, an increase in multidrug-resistant urinary isolates has been reported in recent years[29, 35, 43, 55–58]. Although signs of cystitis often respond well to a course of antimicrobial drugs, microbiological cure (e.g. resolution or bacteriuria independent of clinical signs) may not be achieved. In a recent study, despite resolution of signs in most dogs, a course of cephalosporin or trimethoprim sulfa antibiotics led to a microbiologic cure in as few as 50% of treated dogs[59]. This highlights the limitations of routine urine culture; further characterization of the urinary microbiota has the potential to reveal beneficial microbial taxa and their associated functions.

Manipulation of the microbiome may offer an alternative to the use of antimicrobial drugs for the treatment of disease states impacted by dysbiosis. Powerful examples of this type of therapy are the use of fecal microbiome transfers (FMT) for nosocomial *Clostridium difficile* infections and the administration of *Lactobacillus crispatus* vaginal suppositories in premenopausal women with a history of recurrent UTI. In patients with nosocomial *C*. *difficile* infections, administration of FMT results in a “reset” of the GM producing as high as a 79% cure rate[60, 61]. In premenopausal women, the use of *L*. *crispatus* vaginal suppositories immediately after completing a full course of antibiotic therapy for UTI had approximately a 50% reduction in the recurrance of UTI in UTI-prone women[27]. A similar UM manipulation may be useful in treatment or prevention of UTI in dogs. Even though the UM has not been exhaustively described, this idea has already gained support through the purposeful administration of non-pathogenic urinary microbes such as *E*.*coli* strain 83972[39, 62]. Although not yet sucessful, this idea has also been explored, albeit in a limited fashion, in dogs[63].

## Conclusion

The canine urinary bladder is not a sterile environment but rather has its own unique, diverse and rich bacterial microbiota that is unique from the genital and GM, yet conserved between sexes. The canine urinary microbiome is predominated by OTUs in the phylum *Proteobacteria*. These taxa included *Pseudomonas* sp., *Sphingobium* sp., *Acinetobacter johnsonii*, and unclassified (UC) microbes in the families *Bradyrhizobiaceae* and UC family *Xanthomonadaceae*. While unique, there is considerable overlap observed between the genital and urinary microbiota as both shared *Pseudomonas* sp. and *Acinetobacter* sp. as the most abundant taxa. To the authors’ knowledge, this study is the first to characterize the microbiome of the canine urinary bladder, a niche previously considered to be sterile, using culture independent techniques. These findings provide a background for future studies aimed at detecting a potential role of these microbes in health and disease, and perhaps in manipulation of the UM to prevent or treat urinary disease.
